# Prevalence and factors related to secondhand smoke exposure among secondary school-going adolescents in Malaysia: Findings from Malaysia Global Health School Survey 2012 and 2017

**DOI:** 10.18332/tid/136029

**Published:** 2021-06-15

**Authors:** Kuang Hock Lim, Sumarni Mohd Ghazali, Hui Li Lim, Yoon Ling Cheong, Chee Cheong Kee, Pei Pei Heng, Tsye Yih Tiunh, Mohd Hazilas Mat Hashim, Jia Hui Lim

**Affiliations:** 1 Institute for Medical Research, National Institutes of Health, Kuala Lumpur, Malaysia; 2 Clinical Research Centre, Hospital Sultan Ismail, Johor Bahru, Malaysia; 3 Biostatistics and Data Raspatory Sector, National Institutes of Health, Shah Alam, Malaysia; 4 Department of Pharmacy and Pharmaceutical Sciences, Monash University Malaysia, Subang Jaya, Malaysia

**Keywords:** Malaysia Global Health School Survey, school-going adolescents, secondhand smoke

## Abstract

**INTRODUCTION:**

Secondhand (SHS) smoke exposure has caused various health problems. Therefore, continuous monitoring of SHS exposure is important to determine the efficacy of various anti-tobacco measure implemented. The study aims to compare the prevalence and factor(s) associated with SHS exposure among secondary school-going adolescents in Malaysia during 2012 and 2017.

**METHODS:**

We derived data from the Global School Health Survey (GSHS) 2012 and GSHS 2017, which was carried out in Malaysia using multistage sampling to select representative samples of secondary school-going adolescents. Both surveys used similar questionnaires to measure SHS exposure. Descriptive and multivariate logistic regression was used to determine the prevalence and factors associated with SHS exposure.

**RESULTS:**

Approximately four in ten respondents were exposed to SHS in the past week in both surveys (41.5% in GSHS 2012 and 42.0% in GSHS 2017, respectively). Both surveys revealed a significantly higher SHS exposure among respondents who smoked than among non-smokers and higher among males compared to females. The likelihood of SHS exposure in both surveys was also similar, with a higher likelihood of SHS exposure among smoking adolescents and non-smoking adolescents who had at least one smoking parent/guardian, regardless of their own smoking status. Male adolescents had a higher risk of SHS exposure compared to their female counterparts. Meanwhile, SHS risk also increased with age, regardless of smoking status.

**CONCLUSIONS:**

Our findings suggested that there were no changes in the prevalence of SHS exposure and recorded only a slight change in the factors associated with exposure to SHS among school-going adolescents in Malaysia between the years 2012 and 2017. A more pro-active, extensive and comprehensive programme should be implemented to address the problem of SHS exposure. Parents should be advised to stop smoking or abstain from smoking in the presence of their children, and smoking cessation interventions are necessary for smoking adolescents and their parents.

## INTRODUCTION

Many studies have reported on the health-damaging shown to delay the neurodevelopment of youth effects of exposure to SHS^1-3^. Globally, about 0.6 and is significantly associated with poor academic million deaths were reported due to SHS, one-third were children^4^. SHS exposure has been of which achievement and neurocognitive performance among youth^5^. It is also caused lower respiratory illnesses middle ear disease in children, cough, phlegm, wheeze, and breathlessness among children of school age, ever having asthma, low birthweight, and a lower level of lung function during childhood^6,7^. Furthermore, SHS is also associated with an increased risk of middle ear effusion, adenoidectomy or tonsillectomy, atopyimmunoglobulin E-mediated allergy^6,7^, obstructive sleep apnea (OSA) among children and with single or multiple allergic morbidity^8^ and depressive symptoms^9^. Besides effects on health, SHS exposure also increases non-smokers susceptibility to smoking and reduces the likelihood of smoking cessation among young smokers^10^. Therefore, reducing SHS exposure are among the objectives stipulated in the national strategic plan for tobacco control in Malaysia^11^.

In line with the objectives of the National Anti-Tobacco Strategic Plan and Article 8 of the Framework Convention on Tobacco Control (FCTC), which was ratified by the Malaysian government in 2005^11^, the Malaysian Ministry of Health has enacted the Control of Tobacco Products Regulations (CTPR) 2004^12^ which includes the provision of non-smoking areas in public places^13^. The CTPR 2004 has been amended several times to add more non-smoking areas. To date, more public areas have been gazetted as non-smoking areas^13^, among others, in: restaurants, workplaces with a centralized air-conditioning system; health, education, government and cultural facilities; and indoor stadiums. The Ministry of Health has also cooperated with the state governments to introduce the concept of non-smoking areas in selected localities, smoke-free Melaka (Melaka Bebas Asap Rokok, MBAR), smoke-free Penang (PBAR), smoke-free Terengganu (TBAR) and Kelantan smokefree initiative (IKBAR)^14^. In addition, community interventions such as KOSPEN (Komuniti Sihat, Pembina Negara – Healthy Community, Developed the Nation) were launched in 2014 to address the higher prevalence of NCD risk factors which include encouraging the community to quit smoking and creating a smoke-free home^15^, in addition to health promotion activities in schools to provide awareness to youths about the dangers of tobacco smoke and legislation of smoke-free areas to protect non-smokers from exposure to SHS. Apart from that, the increase in smoking cessation activities among adults through a partnership of public and private practitioners would ensure Malaysia can achieve tobacco end-game by 2045^11^.

The Global School Health Survey conducted in Malaysia in 2012 among secondary school-going adolescents reported 4 out of 10 respondents were exposed to SHS in the past week^16^. The study found that the prevalence and likelihood of SHS exposure were higher among males, older adolescents, Malay and other Bumiputra ethnic group smokers, and adolescents who had smoking parents^16^. But measures implemented since 2012 such as community intervention programs^15^, expansion of non-smoking areas^14^, raising awareness of SHS through health promotion programs organized by Ministry of Health, Malaysia (MOH) agencies and non-government organization (NGOs), and so on, may have changed the pattern of SHS exposure among adolescents^15^. Therefore, this study aims to describe the prevalence and factors related to exposure to SHS among secondary school-going adolescents in 2017 in comparison to 2012.

## METHODS

We derived data from the GSHS 2012 and GSHS 2017 surveys. Both surveys were cross-sectional, nationwide, school-based, and employed multistage sampling to select representative samples of the secondary school-going adolescents in Malaysia. The first stage of sampling was the stratification of each state in Malaysia, followed by the division of school locality by urban and rural areas for each state. The primary sampling units (schools) were selected in each state based on proportionate-to-size sampling, and three classes were randomly selected from each selected school by simple random sampling. All students in the selected classes were invited to participate in the study. The Ministry of Education (Malaysia) and State Education Department approved the protocol of the study, and the Medical Research Ethical Committee, Ministry of Health, Malaysia and the Ethical Committee of MOE have granted the ethical clearance for both surveys.

The active consent approach was used in both surveys to obtain permission from the respondents’ parent or guardian. The consent form and an accompanying letter were distributed through the school administration to the family, and the parents/guardian were informed of the objective of the study as well as the terms and conditions of participation in the study^17^. Only parents/guardian who consented were asked to return the filled form to the school administration. Prior to data collection, trained research team members have conducted a briefing session for the respondents to explain the objectives of the survey, the items in the questionnaire, their right to refuse to answer any of the questions, and the preservation of anonymity. Meanwhile, respondents have also fill in a consent form.

Similar items and definitions were used in both surveys. The dependent variable (exposure to SHS) was measured using the item: ‘During the past seven days, on how many days did people smoke in your presence?’. Students who responded ‘0 days’ were categorized as ‘Not exposed to SHS’, while those who answered ‘1–2 days’, ‘3–4 days, ‘5–6 days’ or ‘All 7 days’ as exposed to SHS. The independent variables in both surveys are gender, age-group (13–15 years or ≥16 years), smoking status (current smokers – smoked at least once in the last 30 days), parental smoking status (neither, either mother or father, both parents), and ethnicity (Malay or other).

### Data management

The data for the study were cleaned and weighted (taking into account the study design and poststratification weight). The characteristics of the respondents were expressed in percentages, and 95% confidence intervals were used to determine the significant differences in distribution by sociodemographic characteristics between both surveys. Chi-squared analysis was employed to test the associations between independent variables and the dependent variable (exposure to SHS). The independent variables in bivariate analysis with a p-value ≤0.25 were included in a multivariable logistic regression model. All possible two-way interactions between the independent variables were checked. Significant interactions were found between gender and smoking status, smoking status and age, smoking status and parental smoking status. Hence, the multiple logistic regression model was stratified by smoking status and two separate models generated for each survey, one for smokers and another for nonsmokers. All statistical analyses were carried out at the 95% significance level using the complex samples module in SPSS statistics software version 20^18^.

## RESULTS

The total number of respondents was 28758 and 30000 in GSHS 2012 and GSHS 2017, respectively. The response rates for GSHS 2012 and GSHS 2017 were 88.7% (25507) and 89% (27497), respectively. The sociodemographic characteristics of respondents of the two surveys (gender, age group and ethnicity) were similar. The proportion of gender for both surveys was approximately equally distributed, with approximately 6 in 10 respondents aged 13–15 years and of Malay ethnicity. However, the proportion of current smokers and those who have at least one parent who smokes were significantly higher among GSHS 2017 survey respondents (Table 1).

**Table 1:** Sociodemographic characteristics and parent/guardian smoking status of GSHS 2012 and GSHS 2017 respondents

*Characteristics*	*GSHS 2012*			*GSHS 2017*		
	*Estimated population*	*Sample*	*% (95% CI)*	*Estimated population*	*Sample*	*% (95% CI)*
**Gender**						
Male	1126613	12732	50.2 (49.4–50.9)	1064953	13135	49.6 (48.9–50.4)
Female	1118911	12729	49.8 (49.1–50.6)	1081492	14362	50.4 (49.6–51.1)
**Age** (years)						
13–15	1399022	16361	62.4 (60.5–64.8)	1302499	16952	60.7 (59.9–61.4)
≥16	847958	9086	37.4 (35.2–39.5)	843946	10545	39.3 (38.6–40.1)
**Ethnicity**						
Malay	1366133	17086	60.8 (56.5–64.9)	1354538	18713	63.1 (62.4–63.8)
Other	880085	8378	39.2 (35.41–43.5)	791905	8784	36.9 (36.2–37.6)
**Smoking status**						
Yes	269079	2836	11.5 (10.4–12.7)	295663	3595	13.8 (13.3–14.2)
No	1981135	22671	88.0 (87.5–88.5)	1850094	23892	86.2 (85.7–86.7)
**At least one parent/guardian smoked**						
Yes	901410	10262	40.2 (39.5–40.9)	865381	11083	43.6 (42.8–44.4)
No	1340356	15150	59.8 (59.1–60.5)	1120832	14510	56.4 (55.6–57.2)

Table 2 shows a significantly higher proportion of exposure to SHS among males, current smokers, at least one parent smokes, Malay ethnic, and aged ≥16 years, in both surveys. A comparison of the SHS exposure for both surveys showed that SHS exposure was significantly lower among current adolescent smokers, 74.1%, (95% CI: 72.3–75.9) in 2017 versus 86.9% (95% CI: 84.7–88.2) in 2012. However, the proportion of SHS exposure was significantly higher among respondents with at least one smoking parent in 2017 compared to 2012 (60.3%, 95% CI: 59.1–61.4 vs 56.3%, 95% CI: 54.4–58.2).

**Table 2:** Prevalence of secondhand smoke (SHS) among school-going adolescents by sociodemographic characteristics, and parent and respondents smoking status

*Variable*	*GSHS 2012*			*GSHS 2017*		
	*Estimated population*	*Sample*	*% (95% CI)*	*Estimated population*	*Sample*	*% (95% CI)*
**Gender**						
Male	560874	6257	50.0 (47.7–52.3)	524065	6435	49.3 (48.2–50.4)
Female	371577	4345	33.2 (31.6–34.9)	376495	4950	34.9 (33.8–37.9)
**Age** (years)						
13–15	506121	6007	36.4 (34.8–38.1)	483998	6203	37.2 (36.3–38.1)
≥16	427291	4607	50.0 (47.5–52.6)	416561	5182	49.4 (48.2–50.7)
**Ethnicity**						
Malay	647575	7926	47.5 (45.2–49.8)	609364	8278	45.1 (44.1–46.0)
Other	285271	2677	32.5 (30.3–34.8)	291196	3107	36.8 (35.6–38.1)
**Smoking status**						
Yes	221972	2360	86.9 (84.7–88.7)	218363	2663	74.1 (72.3–75.9)
No	705562	8186	35.7 (34.1–37.3)	682197	8722	36.9 (36.8–37.7)
**At least one parent/guardian smokes**						
Yes	506428	5784	56.3 (54.4–58.2)	521306	6646	60.3 (59.1–61.4)
No	363071	4169	30.1 (28.4–31.9)	314075	3934	28.0 (27.1–29.0)

Multivariable analysis showed similar odds of SHS exposure among non-smoking adolescents in the two surveys, i.e. significantly higher among males, aged ≥16 years, Malay ethnic, and had at least one parent who smoked. However, no similar pattern was observed of smoking adolescents, in which the odds of exposure were higher among smokers aged ≥16 years and those with a smoking parent in both surveys. In 2017, the odds of SHS exposure were increased for male smokers, and smokers and non-smokers with at least one parent who smoked; however, the odds of SHS exposure were lower among Malays (Table 3).

**Table 3:** Multiple logistic regression for the association between sociodemographic factors and SHS exposure in the GSHS-M 2012 and 2017

*Variable*	*GSHS 2012*		*GSHS 2017*	
	*Smokers (n=2355) AOR (95% CI)*	*Non-smokers (n=21464) AOR (95% CI)*	*Smokers (n=2355) AOR (95% CI)*	*Non-smokers (n=21464) AOR (95% CI)*
**Gender**				
Male	1.50 (0.97–2.33)	1.64 (1.52–1.77)	4.46 (3.45–5.75)	1.51 (1.40–1.62)
Female (Ref.)	1	1	1	1
**Age** (years)				
13–15 (Ref.)	1	1	1	1
≥16	2.32 (1.55–3.48)	1.77 (1.61–1.96)	2.22 (1.75–2.82)	1.83 (1.69–1.98)
**Ethnicity**				
Malay	1.76 (1.21–2.54)	1.72 (1.48–2.02)	1.01 (0.80–1.28)	1.31 (1.21–1.43)
Other (Ref.)	1	1	1	1
**At least one parent/guardian smokes**				
Yes	1.62 (1.14–2.30)	3.03 (2.73–3.33)	3.07 (2.45–3.85)	4.19 (3.88–4.52)
No (Ref.)	1	1	1	1

AOR: adjusted odds ratio. Ref.: reference.

## DISCUSSION

### Prevalence of exposure to SHS

The study revealed that SHS exposure in 2017 was 42.0%, not significantly different from the prevalence in 2012^16^. However, this prevalence is lower than in two other Malaysian studies, i.e. 10%^19^ and 16% lower^20^. In addition, this figure is also lower than the prevalence reported among middle and high school students in Kuwait (45.8% and 51.6%, respectively)^21^. Also, Xi et al.^22^ have reported a prevalence of 55.9% in 68 lower and low-income and middle-income countries, and 67.5% reported among youth in Vietnam^23^. However, the prevalence of SHS is almost equal to the global estimated prevalence of 40% among children^24^ and 42% among youths in the state of Florida in the United States^25^. Gentzke et al.^26^ similarly reported no change in SHS exposure among middle and high school students from 2015 through 2017. This indicates that there has been no change in the smoking pattern in Malaysia during this period^27^ and the anti-tobacco measures that were implemented had not significantly impacted SHS exposure among youths. Due to that, more proactive steps that involve parents, schools and authorities should be formulated to achieve this objective.

### Factor(s) associated with SHS exposure

Smokers were more likely to be exposed to SHS compared to non-smokers in a ratio of 2 to 1 in 2017, which was slightly reduced from 2.43 to 1 in 2012. We postulate that this may be due to a decrease in the percentage of smokers who smoked with other smoking peers, therefore reducing the prevalence of SHS exposure by 12.6%. However, this phenomenon persists among male adolescents, based on multivariate analysis and comparisons made with exposure in 2012. We further hypothesize that decreased smoking with peers is more significant among female smokers as the odds of being exposed to SHS were significantly higher among males in 2017 than in 2012, possibly because female smokers tend to smoke in private, as female smoking is not a norm in Malaysian society. In addition, Malaysia is a communal society, where the views of the society precede individual choice, presumably causing female smokers to smoke individually to avoid public censure^28^. However, this theory needs to be confirmed through subsequent investigation in future studies. The study also found significantly lower prevalence and odds of SHS exposure among non-smokers, possibly because non-smokers have more knowledge on the hazards of SHS^16,29^ and have a negative attitude towards smokers^23,30^; they would therefore tend to avoid SHS exposure.

The odds of SHS exposure were also higher among non-smoking male adolescents, and this was in contrast with the findings of studies among students in Kuwait^21^, Saudi Arabia^31^ and the United States^26^. But, the results of our study are consistent with the findings of other local studies^16,19-20^. This finding may be explained by the higher prevalence of smoking among male adolescents in Malaysia and the tendency of males to mingle with other males; this might cause non-smoking male adolescents more likely to be exposed to SHS from their smoking peers.

Consistent with previous local^16,19,20^, and international studies^22,26,32^, the prevalence and odds of exposure to SHS were significantly higher among older adolescents (i.e. 16 and 17 years old), and this pattern has not changed since 5 years ago. This observation may be explained by the higher prevalence of smoking among adolescents from this age group, and in view of respondents tend to have same-age peers; this will increase the odds of exposure to SHS. In addition, parents and guardians tend to restrict their smoking behavior to protect young children, which diminishes for older children^33,34^. Furthermore, parents and guardians also provide more freedom to the respondents aged ≥16 years compared to those aged 13–15 years. Therefore, they are more likely to patronize more public places such as bus stations, bistros, and therefore, the likelihood for them to be exposed to SHS is high, but our hypothesis needs to be tested in future studies.

Our study found that the prevalence and odds of exposure to SHS were significantly higher among school-going adolescents who have at least one parent/guardian who smokes; this finding is in congruence with the study of Sumarni et al.^16^ and systematic review by Orton et al.^35^. In addition, Park^36^ also reported that concentrations of urine 4-(methylnitrosamino)-1-(3-pyridyl)-1-butanol (NNAL) were highest among children with smoking parents, almost 4 times compared to children with non-smoking parents and no SHS exposure at home (0.996 pg/mg, 95% CI: 1.026–1.427). A comparison made on the prevalence and odds of exposure found a significant increase in 2017 compared to 2012 when one of the parent/guardians smoked. These findings suggested a possible displacement of smoking among adult smokers from public places into the home or into vehicles, although this phenomenon is not supported by the results of Lim et al.^37^ study comparing exposure rates at home between 2011 and 2015. We postulate that this may be a relatively strict anti-smoking policy since 2015, such as the expansion of non-smoking areas in open eateries, which may have led to the findings in this study.

We found that the rate of SHS exposure among adolescents from Malay ethnicity was significantly high compared to their counterpart of non-Malays; the high prevalence of smoking among Malay adults and youth^31,38^ may be a possible explanation for the findings in our study. However, compared to 2012, there was a slight decrease in prevalence and odds of being exposed to SHS among Malay adolescents. This finding is encouraging, where Community Intervention such as KOSPEN implemented mainly in rural areas where the majority of the participants are Malay, and the ‘Smoke-free home’ element in the KOSPEN might have imparted knowledge and encouraged adult or adolescents smokers to smoke outside the house^15^. However, the findings of the lower odds of smokers and non-smokers of adolescents of Malay descent was also contributed by the increased prevalence of SHS exposure among non-Malays. This finding suggests further detail analysis should be carried out on SHS exposure to identify the ethnicity with higher prevalence of SHS exposure, and appropriate measures should be taken to address it.

### Strength and limitations

Our study has several strengths. The GSHS 2012 and GSHS 2017 used standard procedures for selecting participants and the same questions, thus enabling the results between the surveys to be directly compared. Moreover, our study included a national representative large sample size, which enables the generalization of school-going adolescents in Malaysia.

Several limitations should also be noted. First, the use of tobacco products and exposure to secondhand smoke were self-reported, which might not necessarily reflect the true prevalence. However, studies have substantiated the validity of self-reported smoking in young adolescents using the objective measure of urinary cotinine and exhaled CO concentration^39,40^. Also, independent variables that have been shown to be significant in other studies, such as smoking status of household member, smoking status of peer^41,42^, knowledge of SHS health hazard and attitude towards smoking were not investigated^30^. Smoking restriction at home and location of exposure to SHS was also not determined. Therefore, the suitable measures that can be recommended to address SHS exposure in different settings cannot be formulated. Furthermore, there are some variables that show the characteristic differences of respondents that differ between 2012 and 2017 (i.e. smoking status and smoking status by parents) that may affect to some extent the comparisons made.

## CONCLUSIONS

The study showed that SHS exposure among school-going adolescents SHS did not change within the 5-year period between 2012 and 2017. This finding indicates that the measures and policies implemented were still not able to change the rate of SHS exposure. The results might be due to smoking patterns, and related aspects have not changed much in the Malaysian population. Therefore, proactive measures should be taken to reduce exposure to SHS among school-going adolescents. Parents and guardians who smoke should be advised not to smoke in front of their children. Meanwhile, programs should be designed to increase smoking cessation, among parents and respondents who smoke, through counselling or pharmacological assistance. In addition, more stringent enforcement of smoke-free public areas should be implemented by the relevant enforcement agencies. Future studies should include the variables that were identified as related to SHS exposure, frequency and location of SHS exposure, as well as awareness and attitude regarding SHS. Finally, the prevalence of SHS exposure is still high among school-going adolescents in Malaysia, especially among male, current smokers, older age group school-going adolescents, and school-going adolescents with parent(s) who smoked.
